# Adænoid Tumor of Soft Palate

**Published:** 1877-07

**Authors:** W. W. Taylor


### ARTICLE IX.

### *Adœnoid Tumor of Soft Palate.*

On April 14th, 1877, Peter T——, aged thirty; American born; laborer; called to consult Dr. Haywood and myself in regard to a tumor of the soft palate. It is situated to the left of the raphe, is of firm, dense inelastic consistence, movable, the appearance of the mucous membrane over it is normal, and it is about the size and form of a hen's egg. The lower portion extends backward and occludes the posterior nares. The tumor first made its appearance about nine years ago and was slow in its development, attaining nearly its present size within about three years. The past two or three months it has began to grow again and gives the patient great uneasiness. His general health is good and presents a good family history.

April 21st. I removed the tumor, being assisted by Drs. Max and Haywood of this city. The operation performed consisted in making an incision over it and then enucleating, the greatest part of the enucleation being effected with the finger. After the removal of the tumor, the mucous membrane contracted so much, that the edges had to be brought together by silk sutures. No anæsthetic was used. There was very little hemorrhage, the tumor being fed only by capillaries. The soft palate was not lost. The tumor is enclosed in a sac and contains fatty substance, there being very little, if any, connective tissue. It is larger than anticipated before its removal, being near the size of a turkey's egg. The parts healed kindly and in five days after the operation there was no wound at all. As can be easily imagined, it was a great source of annoyance, interfering with his breathing and deglutition. In fact it is difficult to realize that one could live at all with such a large tumor of the soft palate. Hence this case is interesting:

1st. From the unusual locality of the tumor.

2nd. From its unusual large size in that locality, and

3rd. From its remaining the same size for about six years and then suddenly growing.—*W. W. Taylor, M. D., in Nashville Med Journal.*

